# Selection of a Relevant *In Vitro* Blood-Brain Barrier Model to Investigate Pro-Metastatic Features of Human Breast Cancer Cell Lines

**DOI:** 10.1371/journal.pone.0151155

**Published:** 2016-03-09

**Authors:** Aurore Drolez, Elodie Vandenhaute, Sylvain Julien, Fabien Gosselet, Joy Burchell, Roméo Cecchelli, Philippe Delannoy, Marie-Pierre Dehouck, Caroline Mysiorek

**Affiliations:** 1 Université d’Artois (UArtois), EA2465, LBHE, Lens, F-62307, France; 2 Structural and Functional Glycobiology Unit, UMR CNRS 8576, University of Lille, Villeneuve d’Ascq, France; 3 Cell Plasticity and Cancer, U908 INSERM, University of Lille, Villeneuve d’Ascq, France; 4 Research Oncology, King's College London, London, SE1 9RT, United Kingdom; Hungarian Academy of Sciences, HUNGARY

## Abstract

Around 7–17% of metastatic breast cancer patients will develop brain metastases, associated with a poor prognosis. To reach the brain parenchyma, cancer cells need to cross the highly restrictive endothelium of the Blood-Brain Barrier (BBB). As treatments for brain metastases are mostly inefficient, preventing cancer cells to reach the brain could provide a relevant and important strategy. For that purpose an *in vitro* approach is required to identify cellular and molecular interaction mechanisms between breast cancer cells and BBB endothelium, notably at the early steps of the interaction. However, while numerous studies are performed with *in vitro* models, the heterogeneity and the quality of BBB models used is a limitation to the extrapolation of the obtained results to *in vivo* context, showing that the choice of a model that fulfills the biological BBB characteristics is essential. Therefore, we compared pre-established and currently used *in vitro* models from different origins (bovine, mice, human) in order to define the most appropriate tool to study interactions between breast cancer cells and the BBB. On each model, the BBB properties and the adhesion capacities of breast cancer cell lines were evaluated. As endothelial cells represent the physical restriction site of the BBB, all the models consisted of endothelial cells from animal or human origins. Among these models, only the *in vitro* BBB model derived from human stem cells both displayed BBB properties and allowed measurement of meaningful different interaction capacities of the cancer cell lines. Importantly, the measured adhesion and transmigration were found to be in accordance with the cancer cell lines molecular subtypes. In addition, at a molecular level, the inhibition of ganglioside biosynthesis highlights the potential role of glycosylation in breast cancer cells adhesion capacities.

## Introduction

Breast cancer is the leading cause of cancer death and the second most common cancer among women worldwide with 1,7 million cases in 2012 (11, 9% of total cancers), according to the World Cancer Research Fund International. In the last decade, with the improvement of therapeutic strategies, breast cancer has a good prognosis when detected at early-stage. However, the occurrence of metastasis is diagnosed in about 30% of breast cancer patients in developed countries [1]. To successfully form metastases, once escaped from the primary tumor, circulating tumor cells have to achieve different sequential steps, from the arrest in the capillary bed of the targeted organ, the interaction with endothelial cells (ECs) and extravasation to reach the new location to form a secondary tumor. Invasion of a given organ depends on specific properties acquired by cancer cells enabling them to preferentially form metastatic tumor deposits in specific organ sites. This preference is called metastatic tropism [2]. Breast cancer cells preferentially form metastases in lung, bone and brain. Brain metastases are diagnosed in 7 to 17% of patients with breast cancer and are generally associated with a poor prognosis; the survival average is four months and the survival rate at one year is less than 20% [3,4]. Some authors reported that a long period of remission usually preceded brain relapse and propose that brain tropism could be acquired by disseminated yet asymptomatic cancer cells during this long disease free period [5]. Such cells would become able to interact and cross the highly specific and restrictive Blood-Brain Barrier (BBB). Moreover, the high rate of mortality associated with brain metastases can be partially explained by resistance to chemotherapy due to the presence of this barrier. The BBB, localized at the level of brain capillary ECs, is a specific and restrictive barrier controlling the exchanges between the blood and the brain tissue in order to maintain the brain homeostasis. The BBB presents a complex and specific architecture where capillary ECs share a split basement membrane with pericytes and are surrounded together by astrocyte end-feet. The BBB belongs, with glial cells and neurons, to the neurovascular unit (NVU). The communications within the different cells of the NVU allowed the differentiation of ECs which acquired restrictive properties to limit and control the access to the brain parenchyma. Specifically, BBB ECs exhibit a network of tight junctions that block the paracellular way preventing passive diffusion of chemicals from the blood to the parenchyma. In parallel, ECs express efflux pumps able to expulse therapeutic compounds like chemotherapy agents back to the blood stream [6]. Based on the fact that metastatic brain tropism could depend on the first interactions between cancer cells and the specific endothelium of the BBB, identification of cellular and molecular mechanisms involved in early interaction steps occurring between these two cell types is an essential requirement.

To study and understand how cancer cells interact with and cross the BBB, there is a crucial need for an adequate *in vitro* system that will model the BBB properties as closely as possible to the physiological structure. Indeed, the process of metastasis formation has been thoroughly investigated using *in vitro* systems but most of them poorly related to *in vivo* mechanisms, mostly due to the origin of the ECs used. Among the well characterized *in vitro* BBB models, most of them are developed using animal cells (mouse, rat, bovine) isolated from brain microvessels. Moreover, the majority of human *in vitro* models, commonly found in cancer literature, uses human umbilical vein endothelial cells (HUVECs) displaying a specific state of differentiation with a limited tightness. In this study, the objective was to identify, a model that exhibits the characteristics closed to the *in vivo* situation and also able to faithfully translate the specific cellular and molecular interactions occurring at the level of the BBB during the initial steps of brain metastases formation, *i*.*e*. the adhesion and migration processes. For that purpose, we compared adhesion capacities of two breast carcinoma cell lines (MCF-7 and MDA-MB-231) representative of two molecular subtypes of breast cancer (luminal and basal-like) to different barrier models [7]. We used (i) well-characterized animal *in vitro* BBB models [8,9], (ii) HUVECS, commonly found in the cancer related literature [10], and (iii) a new human BBB model recently developed in the laboratory [11,12]. To be considered as valuable, the model should display at once BBB properties closed to *in vivo* data and interactions with breast cancer cell lines which fit with their described propensity to form brain metastases. At the molecular level, the involvement of breast cancer glycosylation in adhesion was analyzed.

## Materials and Methods

### Human *in vitro* BBB model: BLECs model

#### CD34+ cells differentiation from human umbilical cord blood

Endothelial cells were derived from CD34^+^-cells from human umbilical cord blood according to the method described by Pedroso *et al*. [13]. It required the collection of human umbilical cord blood: infants’ parents signed an informed consent form, in compliance with the French legislation. The protocol was approved by the French Ministry of Higher Education and Research (CODECOH Number DC2011-1321). All experiments were carried out in accordance with the approved protocol. CD34^+^-cells were cultivated on 1% gelatin-coated 24-wells plates (Corning Inc., New York, USA) in ECM medium (Sciencell, Carlsbad, CA, USA) containing 5% of heat inactivated fetal calf serum (FCS from GIBCO, Life Technology, SAS Saint Aubin, France) and 50 ng/mL of Vascular Endothelial Growth Factor (VEGF from PrepoTech Inc. Rocky Hill, USA). After 5 days, time necessary for CD34^+^-cells adhesion, the medium was changed every two days. After 15–20 days, CD34^+^-cells started to differentiate into ECs. ECs were then trypsinized and cultivated on 1% gelatin-coated 100mm Petri dish in ECM medium containing 5% heat inactivated FCS and 50μg/mL gentamicin (Biochrom AG, Berlin, Germany).

#### Pericyte culture

Pericytes, isolated from bovine brain capillaries, were cultivated in Dulbecco’s Modified Eagle’s Medium (DMEM) supplemented with 20% heat inactivated FCS, 2 mM L-glutamine (Merck Chemicals, Darmstadt, Germany), 50 μg/mL gentamicin. After 2 days, the confluent pericyte culture was dissociated using trypsin-EDTA (EthyleneDiamineTetraAcetic acid) solution (Biochrom AG, Berlin, Germany) and cells were seeded on 12-wells plates (5 x 10^4^ cells/cm^2^). The phenotype of the pericytes was characterised according to Vandenhaute *et al*. 2011 [14].

#### Co-culture

CD34^+^ derived endothelial cells (CD34^+^-ECs) were treated with trypsin-EDTA solution and seeded on Matrigel^™^ (BD Biosciences, San Jose, CA, USA) coated filters (Costar Transwell inserts, pore size 0.4 μm or 3 μm, 12-well format, Corning Inc., New York, USA) (8 x 10^4^ cells/cm^2^). Filters were firstly cultivated alone during 6 days without medium in the lower compartment according to the protocol described by Vandenhaute et al. (2016)[15]. Then, filters were placed above wells containing pericytes. The co-culture medium, ECM supplemented with 5% heat inactivated FCS and 50 μg/mL gentamicin, was changed every 2 days. After 6 days of co-culture, the model was stable and ready for experiment.

### Bovine *in vitro* BBB model

In accordance with the French legislation the animal house of the Université d'Artois get approval from the protecting population departmental directorate under number B62-498-5. In compliance with the new European directive (Directive 2010/63/EU), all the procedures were submitted to the ethics committee (comité d'éthique en expérimentation animale Nord Pas-De-Calais: C2EA 75) and the French Ministry (ministère de l'enseignement supérieur et de la recherche: direction générale pour la recherche et l'innovation) for authorization before starting up the project.

Brain capillary endothelial cells were isolated from the brain tissue of 6-month-old calves purchased from a local slaughterhouse (Zidorvignies, Douai, France). The rats used in the study (strain Sprague-Dawley RjHan) were supplied by Laboratoire Janvier (Le Genest-Saint-Isle, France). The rats were housed in a temperature-controlled pathogen-free room with light from 07:00 to 19:00h (daytime) and had free access to food and water and live in an enriched environment.

#### Bovine Brain Capillary endothelial cells (BBCECs)

Endothelial cells were isolated from bovine brain capillaries as described by Méresse et al. [16] and seeded in 1% gelatin-coated 60mm Petri dishes containing DMEM supplemented with 10% heat inactivated Horse Serum (HS), 10% heat inactivated Calf Serum (CS), 2 mM glutamine and 5 μl/mL of basic fibroblast growth factor (bFGF). This medium was renewed every 2 days.

#### Rat glial cells primary culture

Rat glial cells (GCs) primary cultures were made from Sprague-Dawley newborn rat (Janvier, Le genest Saint Isle, France) cerebral cortices as described by Booher and Sensenbrenner [17]. The removed part of the cerebral cortex was separated of meninges and passed through a nylon sieve in order to extract undifferentiated cells. These cells were seeded in 12-well plates (1.4 x 10^4^ cells/cm2). The culture was maintained in DMEM medium with 10% heat inactivated FCS, 2mM L-glutamine and 5μg/mL gentamicin which was changed twice a week. After 21 days, the culture was stable and consisted of astrocyte (60%), microglial cells and oligodendrocyte (40%) [18].

#### Co-culture

When they reached confluence, BBCECs were treated with trypsin-EDTA, dissociated and seeded (4 x 10^5^ cells/mL) on rat-tail collagen-coated filters (Costar Transwell 0.4 μm, 12-well format). Filters were placed above wells containing rat GCs. The culture medium was the same as the one used for ECs alone and was changed every 2 days. In these conditions, ECs formed a confluent monolayer after 5 days and were used 7 days after confluence. In total, 12 days were required for getting the specific properties of cerebral endothelium [8].

### Murine syngenic *in vitro* BBB model

In accordance with the French legislation the animal house of the Université d'Artois get approval from the protecting population departmental directorate under number B62-498-5. In compliance with the new European directive (Directive 2010/63/EU), all the procedures were submitted to the ethics committee (comité d'éthique en expérimentation animale Nord Pas-De -Calais; C2EA 75) and the French Ministry (ministère de l'enseignement supérieur et de la recherche: direction générale pour la recherche et l'innovation) for authorization, were approved and referenced under the number 2015090115412152.

Mice (C57Bl6/J) were supplied by Laboratoire Janvier (Le Genest-Saint-Isle, France) and housed in a temperature-controlled pathogen-free room with light from 07:00 to 19:00 h (daytime) and had free access to food and water and live in an enriched environment.

#### Mouse brain capillary endothelial cells culture

Endothelial cells were extracted from mice brain microvessels using the method described by Coisne *et al*. [9] and seeded on Matrigel^™^-coated filters (Costar Transwell 0.4 μm, 12-well format). All experiments were performed within the framework of the French legislation that controls animal experimentation. Cells were cultivated until confluence in DMEM medium 5% heat inactivated CS, 2 mM L-glutamine, 50 μg/mL gentamicin and 1 ng/mL bFGF. This medium was changed every day.

#### Mouse glial cells primary culture

Mouse glial cells (GCs) primary cultures were made from C57Bl6/J newborn mouse (Janvier, Le Lenest Saint Isle, France) cerebral cortices as described by Booher and Sensenbrenner [17]. The removed part of the cerebral cortex was separated of meninges and passed through a nylon sieve in order to extract undifferentiated cells. These cells were seeded in 12-well plates (1.4 x 10^4^ cells/cm^2^). The culture was maintained in DMEM medium with 10% heat inactivated FCS, 2mM L-glutamine and 5μg/mL gentamicin which was changed twice a week. After 21 days, the culture was stable and consisted of 84% of astrocytes, 10% of oligodendrocytes and 6% of microglial cells [9].

#### Co-culture

The co-culture, started 24 hours after the seeding of ECs on filters, was performed in the same medium as for ECs alone and was changed every day. ECs form a confluent monolayer and were used for experiment after 5 days.

### Human Umbilical Vein Endothelial Cells (HUVECs) culture

#### HUVECs culture

HUVECs were cultivated in 1% gelatin-coated 100 mm Petri dish containing ECM medium (Sciencell, Carlsbad, CA, USA) with 2% heat inactivated FCS and 50 μg/mL gentamicin. Medium was changed every 2 days.

#### Co-culture

When the HUVECs culture reached the confluence, cells were treated with trypsin-EDTA, seeded onto Matrigel^™^-coated filters (Costar Transwell 0.4 μm, 12-well format) (8 x 10^4^ cells/cm2) and put above wells containing or not pericytes or rat GCs. Medium was changed every 2 days; cells were used for experiment after 6 days of co-culture.

### Human Breast cancer cell lines culture

Breast cancer cell lines MDA-MB-231 (HTB-26, ATCC^®^, Manassas, VA, USA) and MCF-7 (Catalogue# 86012803, Sigma-Aldrich, St-Louis, MO, USA), ZR-75-1 (CRL-1500, ATCC^®^), Hs 578T (HTB-126, ATCC^®^ kindly provided by Dr. Van Slambrouck, New Mexico Institute of Mining and Technology, NM, USA), BT-20 (HTB-19, ATCC^®^) and SK-BR3 (HTB-30, ATCC^®^) were cultivated in DMEM medium with 4.5 g/L D-glucose, 10% heat inactivated FCS, 2 mM L-glutamine and 5 μg/mL penicillin-streptomycin. Cells were cultivated 3 weeks before being used in adhesion experiment.

### Cancer cells adhesion/transmigration assays

#### Cancer cells staining

In order to visualize cancer cells at the end of the adhesion and transmigration kinetics, the cells were previously loaded with a fluorescent CellTracker^™^ (Invitrogen, Carlbad, USA). The cancer cells were incubated with the compound (diluted at 10 μM in DMEM) during 45 minutes at 37°C. The medium was then removed and after one washing step with DMEM, replaced by complete medium for a minimum of 45 minutes.

#### Adhesion assay

First, cancer cells were treated with EDTA and mechanically dissociated in complete medium containing only 1% heat inactivated FCS. Then, the cancer cells were seeded (2 x 10^4^) on 0.4 μm pore size filters containing ECs monolayer. After 120 minutes, the supernatant was gently removed and filters were rinsed with DMEM. Filters were then fixed with 4% paraformaldhehyde solution for 10 minutes. After the staining of nuclei with Hoechst 33358 (Bis Benzimide, MP Biochemicals, Irvine, CA, USA), the filters were mounted using Mowiol solution containing DABCO (1, 4-Diazobicyclo-(2.2.2-octane)) as an anti-fading agent.

#### Transmigration assay

Using the same protocol, cells were seeded (8x10^4^) on 3 μm pore size filters containing endothelial monolayers. After 16 hours, filters were fixed for 10 min using 4% paraformaldehyde at room temperature, the nuclei were stained with Hoechst 33358 and the filters were placed upside down on glass slides. Samples were finally mounted under coverslips with Mowiol containing DABCO.

#### Chemical inhibition of Glucosylceramide synthase

Breast cancer cell lines were treated during 5 days with 10 μM of PPMP (DL-threo-1-Phenyl-2-palmitoylamino-3-morpholino-1-propanol) (Sigma-Aldrich, St-Louis, USA) added in DMEM medium containing 4.5 g/L D-glucose, 10% heat inactivated FCS, 2 mM L-glutamine and 5 μg/mL penicillin-streptomycin according the protocol described in Cazet et al. 2009 [19]. No cell toxicity was measured upon 5 days of culture (Lactate dehydrogenase assay).

#### Results and statistics

Adherent and transmigrated cancer cells were manually counted on the total surface of each filter under Leica DMR fluorescence microscope (Leica Microsystem, Wetzlar, Germany). In the interest of clarity, the number of adherent or transmigrated MDA-MB-231 on each model was set to 100%. All results were expressed as mean ± SEM from two or more independent experiments. Statistical significance was assessed by t-test. All statistical analyses were performed using GraphPad Prism version 5.0 for Windows (GraphPad Software, San Diego, California, USA).

### Permeability measurement

BBB ECs permeability was measured according to the method described by Dehouck et al., [20]. Diffusion of a hydrophilic molecule, Lucifer Yellow (LY, lucifer yellow CH dilithium salt, Sigma-Aldrich) or [^14^C]-saccharose (1μCi/mL, MM = 342) (Amersham Biosciences, Piscataway, USA) that poorly cross the BBB was measured and used as integrity markers. Filters containing ECs were placed in wells containing 1.5 mL Ringer-Hepes solution (RH) (150 mM NaCl, 5.2 mM KCl, 2.2 mM CaCl_2_, 0.2 mM MgCl_2_6H_2_O, 6 mM NaHCO_3_, 5 mM HEPES, pH: 7.4). After filters transfer, 0.5 mL of RH containing 50 μM of LY or [^14^C]-saccharose at 0,1μCi/mL was added to the upper compartment. After different time points (15, 30, 45 and 60 minutes), filters were placed in a new well containing RH. An aliquot of 200 μL from the lower compartment at each time point and an aliquot of 20 μL from the initial solution of LY (Synergy H1, Thermo Labsystems, Issy-Les-Moulineaux, France) or [^14^C]-saccharose (liquid scintillation counter Packard Tricarb 2100 TR, Perkin Elmer, Courtaboeuf, France) were used for quantification. The endothelial permeability coefficient of LY (Pe^LY^) and [^14^C]-saccharose (Pe^Saccharose^) were calculated as described by Siflinger-Birnboim *et al*. [21]. To obtain a concentration-independent transport parameter, the clearance principle was used. Briefly, the average volume cleared is plotted *versus* time, and the slope is estimated by linear regression. Both insert permeability (PS_f_, for insert only coated with Matrigel^™^ or collagen) and insert plus endothelial cell permeability (PS_t_, for insert with Matrigel^™^ or collagen and cells) were taken into consideration, according to the following formula: 1/PS_e_ = 1/PS_t_− 1/PS_f._

The permeability value for the endothelial monolayer was then divided by the surface area of the porous membrane of the insert (membrane surface 1.12 cm^2^) to obtain the endothelial permeability coefficient (Pe) of the molecule (in cm/min).

### Immunofluorescent staining of endothelial cells

#### Endothelial cells

Cells were fixed in 4% paraformaldehyde for 10 minutes at room temperature and permeabilized with 0.1% saponin 30 min. After several washes in PBS (Phosphate Buffered Saline Calcium) (8 g/L NaCl, 0.2 g/L KCl, 0.2 g/L KH_2_PO_4_, 2.86 g/L NaHPO_4_ (12 H_2_O), pH 7.4), nonspecific sites were blocked for 30 minutes with PBS containing 10% normal goat serum (NGS) (Sigma-Aldrich). Cells are then incubated with primary polyclonal antibody against Claudin-5 (Rabbit anti-Claudin-5, 1/200^e^, 34–1600, Life Technology) or against ZO-1 (Rabbit anti -O-1, 1/200^e^, 61–7300, Life Technology) diluted in PBS 2% NGS, for 1 hour at room temperature. After several washing steps with PBS, the secondary polyclonal antibody (Goat anti-rabbit Alexa 568, A11036, Molecular probes) diluted at 1/200^e^ in PBS 2% NGS, was incubated in the dark for 1 hour. Nuclei are stained with Hœchst 33358 and washed with PBS and distilled water.

#### GM1 staining on breast cancer cells

The effect of PPMP treatment on the inhibition of ganglioside biosynthesis was observed thanks to the staining of the ganglioside G_M1_. Following 5 days with or without PPMP treatment, cancer cells were fixed 10 minutes with paraformaldehyde at room temperature, before blocking the non-specific binding sites with PBS containing 10% of bovine serum albumin (BSA) during 30 minutes at room temperature. After several washing steps with PBS, the anti-G_M1_ antibody conjugated with FITC, diluted at 1/1000^e^ in PBS containing 1% BSA, was incubated during 1 hour at room temperature. After several washing steps with PBS, the nuclei were stained with Hœchst 33358 and washed with PBS.

#### Observation

Preparations were mounted using Mowiol containing an anti-fading agent (DABCO). Cells were observed with a Leica DMR fluorescence microscope. Images were collected using a cool snap photometrics camera (Leica Microsystems) and are processed using Adobe Photoshop software 5.5 (Adobe systems).

## Results

### Animal *in vitro* BBB models

**Fig 1:**
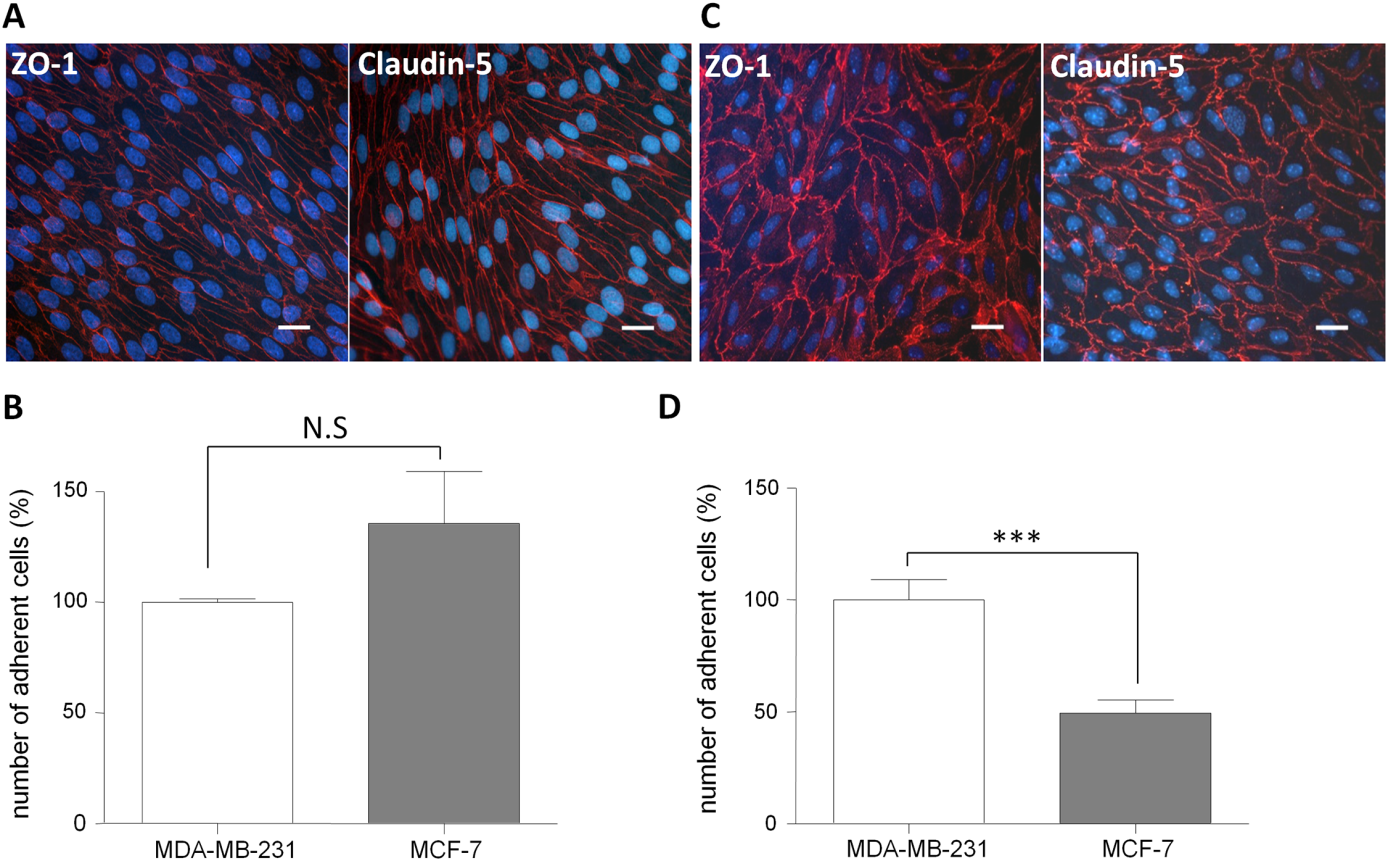
BBB properties and cancer cells adhesion assay on animal *in vitro* BBB models. BBB Integrity measurement. The bovine (A) and the murine (C) models present a continuous staining of tight junction proteins ZO-1 (left panel) and Claudin-5 (right panel) associated with a Pe^LY^ of respectively 0.39 ± 0.07 x 10^−3^ cm/min and 0.36 ± 0.19 x 10^−3^ cm/min. Nuclei are stained with Hoechst (blue), bar = 50 μm. Quantification of cancer cells adhesion. The number of adherent MDA-MB-231 was set up to 100% and equal to 286 for the bovine model (B) and 442 the murine model (D); Pe^LY^: Lucifer Yellow Endothelial Permeability; ZO-1: Zonula-Occludens-1, NS: Non Significant. The results are mean of triplicate and representative of two independent experiments. ***p<0.001.

In order to study the interactions occurring between breast cancer cells and the BBB, in a first step, adhesion abilities of MDA-MB-231 and MCF-7, two human breast cancer cell lines were studied on the well-characterized animal BBB *in vitro* models (Fig 1). The primaries BBCECs and MBCECs were co-cultivated with GCs, during 12 and 5 days respectively, to re-induce BBB properties as previously described [8,9]. These properties were evaluated by measuring the paracellular permeability coefficient of Lucifer Yellow (Pe^LY^) and the immunostaining of tight junction proteins ZO-1 and Claudin-5. Low Pe^LY^ values were obtained on BBCECs and MBCECs monolayers, 0.39 ± 0.07 x 10^−3^ cm/min and 0.36 ± 0.19 x 10^−3^ cm/min respectively. These values were correlated with the presence of continuous immunostaining of ZO-1 and Claudin-5 at the cell junctional borders (Fig 1A and 1C). These results highlighted the expression of restrictive BBB properties by ECs, as expected for a BBB model.

To evaluate cancer cell lines adhesion capacities on the BBB endothelium, the number of adherent cancer cells was quantified after 2h of incubation with the two selected cell lines MDA-MB-231 and MCF-7. On BBCECs, there was no statistically significant difference of breast cancer cells adhesion (Fig 1B). Adherent cancer cells are shown in (S1 Fig). However on the MBCECs model the number of adhered MCF-7 cells is about half as adhered MDA-MB-231 cells (Fig 1D).

Although these animal models present BBB properties, notably, low Pe^LY^ and continuous tight junctions' network, only the MBCECs model allowed to measure a difference of adhesion between MDA-MB-231 and MCF-7 in accordance with their described *in vivo* characteristics.

However, given the actual controversy over the use of animal models, which had been postulated as the common cause of clinical research attrition in central nervous system therapeutic areas [22], we next used models developed with ECs of human origin.

### Human *in vitro* BBB models

The human origin of endothelial cells is required to identify the mechanisms related to the human species purposely at a molecular level. However, to study specifically what happens at the level of the human BBB, the endothelial cells should exhibit specific BBB properties.

**Fig 2:**
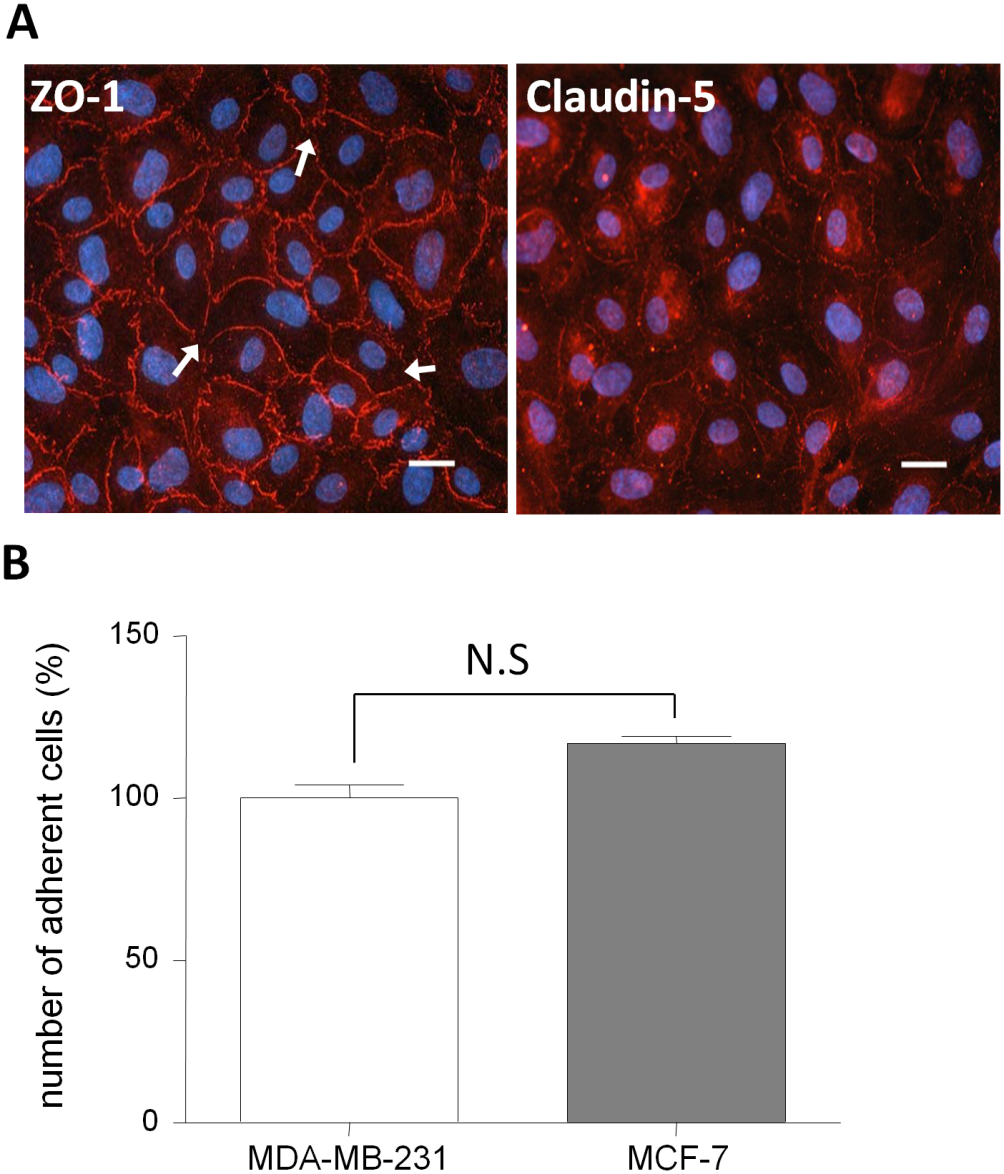
BBB properties and cancer cells adhesion assay on HUVECs *in vitro* model. (A) Integrity measurement. The HUVECs model presents a discontinuous staining of tight junction proteins ZO-1 (left panel) and Claudin-5 (right panel) associated with a high Pe^LY^ of 2.35 ± 0.19 x 10^−3^ cm/min. Interruptions of the staining are indicated by white arrows. Nuclei are stained with Hoechst, bar = 50 μm. (B) Quantification of cancer cells adhesion. The number of adherent MDA-MB-231 was set up to 100% and equal to 1748. HUVECs: Human Umbilical vein endothelial cells; Pe^LY^: Lucifer Yellow Endothelial Permeability; ZO-1: Zonula-Occludens-1. The results are mean of triplicate and representative of three independent experiments. N.S.: Non significant.

First of all, we used an *in vitro* model developed with HUVECs, the most commonly used model in the cancer field to understand *in vitro* the cellular mechanisms involved in the initiation of metastases formation [23,24]. The integrity of the HUVECs layer was analyzed and revealed that after 6 days of culture alone on filters, the Pe^LY^ of HUVECs was 2.35 ± 0.54 x 10^−3^ cm/min. This high Pe^LY^ value was correlated with the discontinuous immunostaining of ZO-1 and Claudin-5 at cell junctions as shown in Fig 2A.

The capacity of interaction of the two breast cancer cell lines with human endothelial cells was analyzed by adhesion experiment and revealed an inverse difference of adhesion on HUVECs where MCF-7 adhere 10% more than MDA-MB-231 (Fig 2B). Adherent cancer cells are shown in (S1 Fig).

Despite the human origin of these ECs, the lack of BBB properties could not reflect the expected interactions that occur between cancer cells and BBB endothelium during the brain metastases formation.

To take into account the cerebral environment involved in the differentiation of the ECs towards a BBB phenotype, HUVECs were co-cultivated with cells from the NVU: Glial cells (GCs) [5] or pericytes (S2A and S2B Fig). Co-culture with GCs does not induce restrictive BBB properties establishment in HUVECs revealed by high Pe^LY^ value (3.92 ± 1.04 x 10^−3^ cm/min) and discontinuous tight junction protein staining (S2A Fig). Conversely, the co-culture with pericytes induced a Pe^LY^ decrease compared with solo-culture. After co-culture, HUVECs showed a Pe^LY^ equal to 0.96 ± 0.12 x 10^−3^ cm/min. In connection with these Pe^LY^ values, HUVECs showed an immunostaining of tight junction proteins but still presenting some gaps (S2B Fig). In conclusion, despite the improvement of barrier properties of HUVECs in co-culture with pericytes, the HUVECs models (solo cultivated and co-cultivated) cannot be used as reliable BBB models.

**Fig 3:**
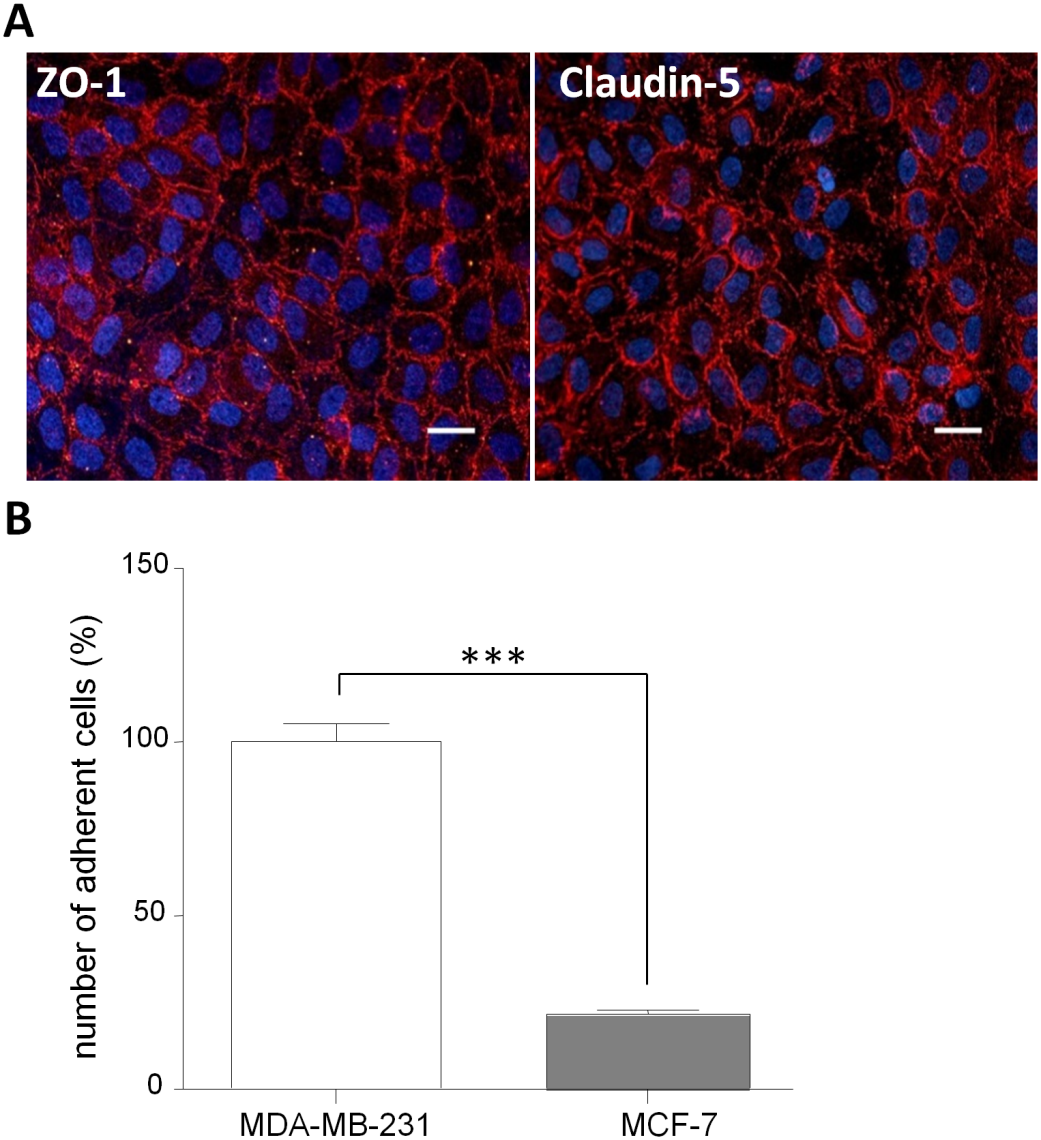
BBB properties and cancer cells adhesion assay on human BBB *in vitro* model. (A) Integrity measurement. The BLECs model presents a continuous staining of tight junction proteins ZO-1 (left panel) and Claudin-5 (right panel) associated with a low Pe^LY^ of 0.58 ± 0.07 x 10^−3^ cm/min. Nuclei are stained with Hoechst, bar = 50 μm. (B) Quantification of cancer cells adhesion. The number of adherent MDA-MB-231 was set up to 100% and equal to 706. BLECs: Brain Like Endothelial Cells; Pe^LY^: Lucifer Yellow Endothelial Permeability; ZO-1: Zonula-Occludens-1. The results are mean of triplicate and representative of six independent experiments. The results are mean of triplicate and representative of three independent experiments. ***p<0.001.

Recently a human BBB *in vitro* model was developed using ECs differentiated from umbilical cord blood CD-34^+^stem cells. Following a co-culture with pericytes which induce the differentiation of the ECs towards a BBB phenotype, these cells display a Pe^LY^ value of 0.58 ± 0.07 x 10^−3^ cm/min. In connection with this Pe^LY^ value, ZO-1 and Claudin-5 stainings were continuous at the cell junctions (Fig 3A). The BBB model obtained after co-culture of CD34^+^-ECs with pericytes, called BLECs (Brain-Like Endothelial Cells) model [11,12].

The adhesion experiment using this model revealed that adhesion of MCF-7 was much lower than MDA-MB-231 adhesion (78% less than the MDA-MB-231) in accordance with their described *in vivo* characteristics (Fig 3B). Adherent cancer cells are shown in (S1 Fig).

Among the human *in vitro* barrier models, the BLECs model is the only one to display expected BBB properties in terms of permeability correlated with a continuous tight junction network and reveal a significant difference of adhesion between the two cancer cell lines.

Among all the *in vitro* models used, the BLECs model is the only one to display at the same time the expected BBB properties, the difference of adhesion of the two cancer cell lines and allow the identification of mechanisms specific of the human species (Table 1).

**Table 1:** Comparative results obtained for BBB characteristics and cancer cells adhesion.

		Essential BBB properties			Valuable BBB model?	Differential of adhesion with cancer cells
		Permeability (10^−3^cm/min)	Tight junction staining pattern			
			Claudin-5	ZO-1		
**Animal models**						
**Mouse**	Primary capillary ECs in coculture with GCs	0,36 ± 0,19	Continuous	Continuous	✓	MDA>MCF-7
**Bovine**	Primary capillary ECs in coculture with GCs	0,39 ± 0,07	Continuous	Continuous	✓	MDA = MCF-7
**Human models**						
**HUVECs**	Umbilical vein ECs	2,35 ± 0,54	Not detectable	Discontinuous	✗	MDA = MCF-7
	Umbilical vein ECs in coculture with pericytes	0,96 ± 0,12	Discontinuous	Discontinuous	✗	N.D
	Umbilical vein ECs in coculture with GCs	3,92 ± 1,04	Not detectable	Discontinuous	✗	N.D
**BLECs**	CD34^+^ (stem cells) derived ECs in coculture with pericytes	0,58 ± 0,07	Continuous	Continuous	✓	MDA>MCF-7

The table summarize the results obtained concerning the analysis of BBB properties (permeability and tight junction staining pattern) and results of breast cancer cells adhesion. ECs: endothelial cells; GCs: Glial cells; HUVECs: human umbilical vein endothelial cells; BLECs: brain like endothelial cells.

### Does the human *in vitro* BBB model able to translate the *in vivo* interaction heterogeneity

**Fig 4:**
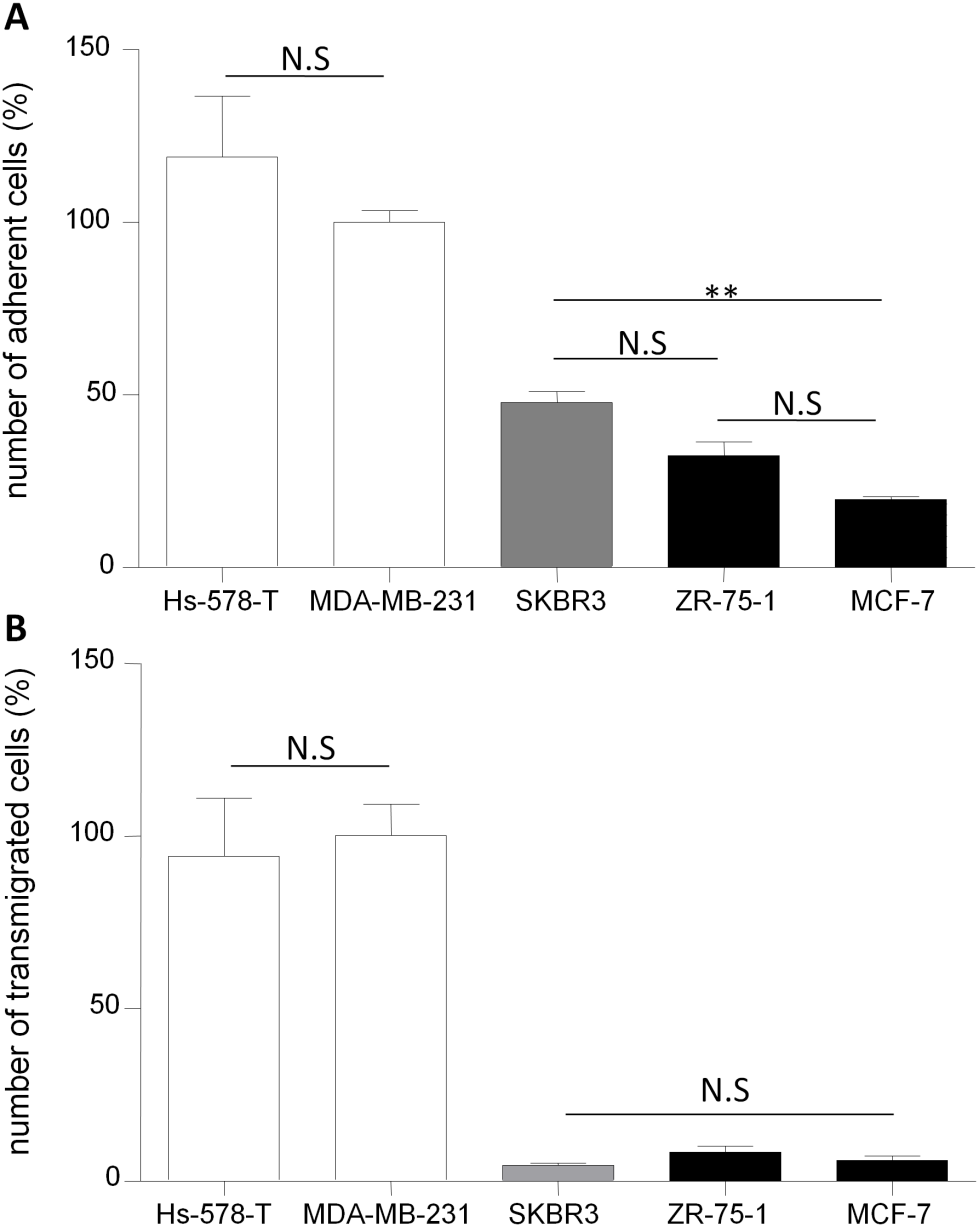
Adhesion-transmigration analysis of breast cancer cell lines representative of different molecular cancer subtypes on the BLECs model. Quantification of cancer cells adhesion. (A) The number of adherent MDA-MB-231 was set up to 100% and equal to 672. The results are mean of triplicate and representative of three independent experiments. Quantification of cancer cells transmigration. (B) The number of transmigrated MDA-MB-231 was set up to 100% and equal to 251. The results are mean of triplicate and representative of two independent experiments. N.S: Non Significant; **p<0.01; ***p<0.001. Basal-like (Triple negative) molecular subtype (open bars), luminal molecular subtype (filled bars).

In order to analyze the behavior of the BLECs model regarding breast cancer cell lines representative of different molecular subtypes of breast cancer, adhesion experiments were done using additional cell lines. Several breast cancer cell lines were chosen according to their described propensity to form brain metastases. The results presented in Fig 4A revealed that MDA-MB-231 and Hs 578T, which belong to the highly metastatic triple negative (basal-like) breast cancer subtype, have the a similar high rate of adhesion with the BBB ECs. Moreover, the same low level of adhesion is measured between the ZR-75-1 and the MCF-7 which both belongs to luminal breast cancer subtype. At least, the SKBR3 cell line described as having an intermediate metastatic behavior between the triple negative and the luminal subtypes breast cancers, adhere to the BBB ECs at the level of 50% of what was measured with the triple negative breast cancer. To summarize, the same results of adhesion were measured for the cell lines representative of the same molecular subtype of cancer.

The BLECs model was used for studying the transmigration which represent the next interaction step for cancer cells to reach the brain parenchyma. To avoid the migration of endothelial cells in the lower face of the 3μm pore size filter, the BLECs models was cultivated according to the protocol described by Vandenhaute et al.2016 [15]. The results indicate that all the lines were able to cross the BBB. Moreover the cell lines presented a differential of transmigration with a rate similar to the adhesion results capacities *i*.*e*. MCF-7 and ZR-75-1 transmigration rate were much lower than for MDA-MB-231 and Hs 578T (84% less than the MDA-MB-231) (Fig 4B). The transmigration rate of SKBR 3 cell line is equivalent of the MCF-7 cell line. The transmigration of all the cell lines did not induced any breakdown of the endothelial monolayer as revealed by the absence of modulation of the endothelial permeability (Pe ^saccharose^ = 0.74 ± 0.03 x 10^−3^ cm/min, n = 27) compared to the control condition before transmigration (Pe ^saccharose^ = 0.72 ± 0.02 x 10^−3^ cm/min, n = 36). The results highlight that the adhesion- transmigration capacities of breast cancer cells are in line with their described propensity to from brain metastases and also the molecular subtypes of cancer to which they belong. The BLECs model is able to translate *in vitro* the *in vivo* heterogeneity of interactions occurring during the interaction process with the different molecular subtypes related-breast cancer cells.

### Effect of the inhibition of ganglioside biosynthesis on breast cancer adhesion capacities to BLECs

**Fig 5:**
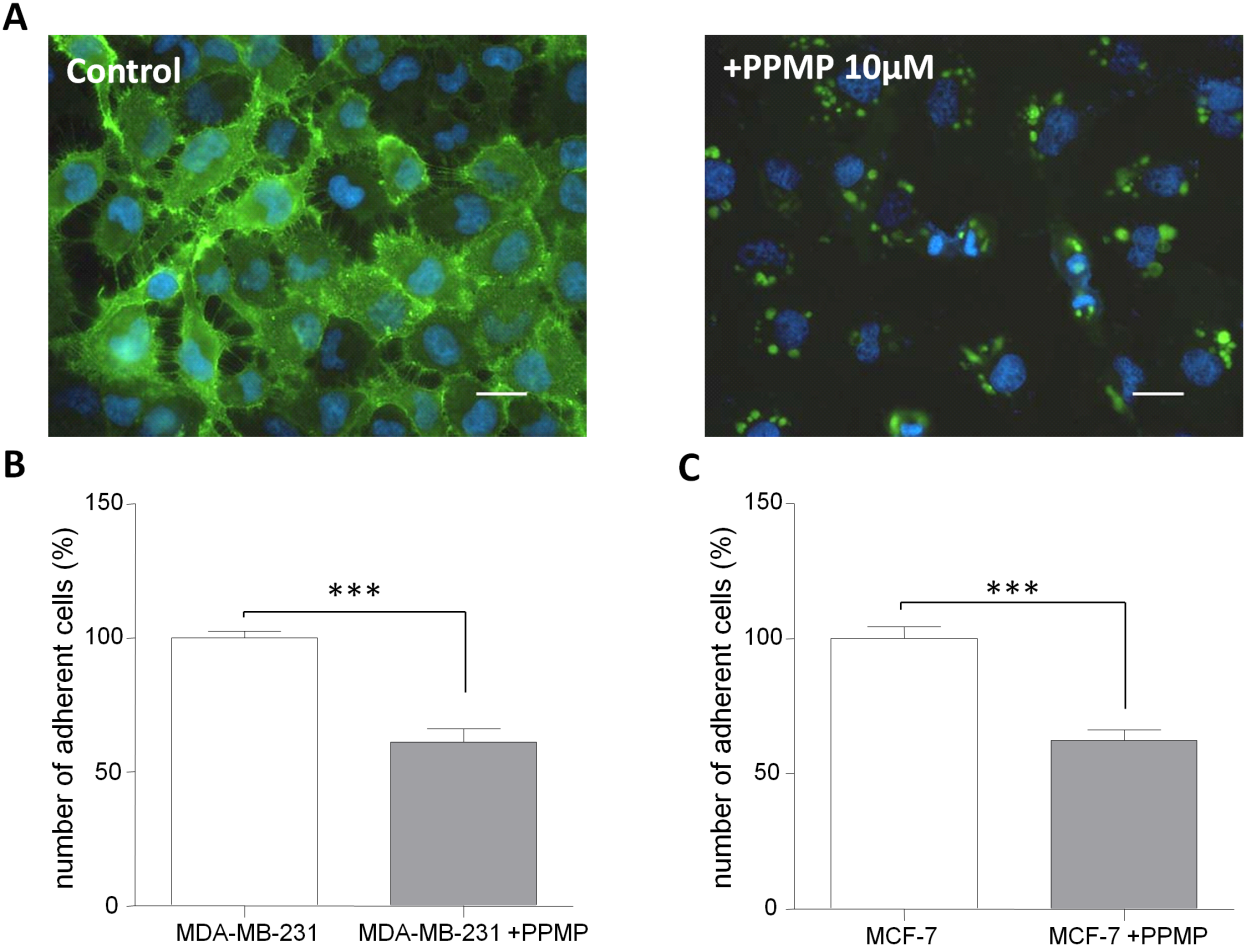
Adhesion analysis of breast cancer cell lines after glycosphingolipid biosynthesis inhibition. (A) Visualization of glycosphingolipid synthesis inhibition. In control condition, MDA-MB-231 expressed the glycosphingolipid G_M1_ (green) at the cell surface (left panel). After 10 μM PPMP treatment during 5 days, G_M1_ expression at the cell surface was significantly reduced (right panel). Nuclei are stained with Hoechst, bar = 50 μm. (B) Quantification of MDA-MB-231 cells adhesion after PPMP treatment. The number of adherent non-treated MDA-MB-231 was set up to 100%. (C) Quantification of MCF-7 cells adhesion after PPMP treatment. The number of adherent non-treated MCF-7 was set up to 100%. The results are mean of triplicate and representative of three independent experiments. ***p<0.001.

The glycosylation is one of the most important modifications of proteins and lipids. Glycoconjugates at the cell surface are thought to play an important role in biological functions and are described as being involved in numerous processes such as cell-cell interaction, cell adhesion and cell differentiation. To identify the involvement of glycosphingolipids in adhesion capacities of breast cancer cells to BBB ECs, their biosynthesis was inhibited using D-L-threo-1-phenyl-2-palmitoylamino-3-morpholino-1-propanol (PPMP), a chemical glucosylceramide synthase inhibitor [19]. As shown in Fig 5A, the expression of the glycosphingolipid G_M1_, visualized by immunofluorescence staining, was reduced in breast cancer cells compared to the non-treated condition.

Adhesion experiment, after PPMP treatment, revealed that both MDA-MB-231 and MCF-7 adhere at a lower rate compared to the non-treated condition (Fig 5B and 5C, respectively). The adhesion capacities are decreased in both cell lines of about 40%.

This result highlights a potential role of breast cancer cells glycosylation in the adhesion process.

## Discussion

Despite the improvement of therapeutic strategies, the treatment of cancer brain metastases remains challenging. Even if the resort to radiosurgery or surgical resection of the brain tumor may provide an additional few months of survival [25,26], those procedures are highly hazardous and far from being satisfying. Thus, brain metastases are still mostly incurable. To form brain metastases, the circulating cancer cells need to extravasate across the brain capillary bed and colonize the brain parenchyma. At the level of brain capillaries, the presence of the BBB limits the exposure of the parenchyma, and consequently the cancer cells, to circulating drugs which could explain the lack of efficiency of chemotherapy treatments. Consequently, it is of prime importance to prevent cancer cells reaching the brain parenchyma.

The formation of brain metastasis is widely analyzed using *in vivo* approach. As no model of spontaneous brain metastasis has been developed, experimental approaches study the propensity of cancer cells to develop metastases in the brain or different organs following intravenous or intracardiac inoculation in mice [27]. However to easily investigate the cellular and molecular mechanisms occurring at the level of the BBB an *in vitro* approach is required. In this context, our study has focused on the first interactions that occurred between breast cancer cells and brain vasculature during adhesion and transmigration of cancer cells to the BBB endothelium. Our aim was to identify a model that enables the study of early interactions occurring during the formation of brain metastases and relevant to human *in vivo* situation.

Firstly, two human breast carcinoma cell lines were used in this study. On one hand, the MDA-MB-231 cell line, representative of the triple negative basal-like breast cancer subtype, which have a high rate of brain metastasis, was used as an aggressive cell line. On the other hand, MCF-7 cell line, was selected belonging to luminal breast cancer subtype, less aggressive and which rarely develops brain metastases in animal [7]. Although both cell lines are derived from metastatic cells in pleural effusion fluid, their behavior *in vitro* and in mouse models shows them to be very different in their aggressiveness. Based on the hypothesis that cancer cells with different brain metastasis properties will exhibit different interaction capacities with the BBB, we have compared adhesion of these two cell lines to *in vitro* endothelial barrier models.

Several *in vitro* BBB models are currently used, each with their own strengths and weaknesses and the choice is firstly dependent on the application. To be considered as a BBB model, the endothelial cells should at least exhibit basic and essential BBB characteristics such as the formation an endothelial monolayer, and display a restricted permeability to BBB integrity marker associated to a continuous network of tight junction proteins [6,28]. Well-characterized and reliable *in vitro* BBB models are animal models produced from mice, rat or bovine cells where primary culture of BBB ECs are isolated from cerebral microvessels. These primary endothelial cells cultures, once extracted, are then co-cultivated with GCs in order to maintain their BBB phenotype [8,9].

In our study, the two well characterized animal BBB models demonstrated different results. The bovine model did not provide any statistically significant adhesion results, probably due to the difference of species between the human cancer cells and the bovine BBB ECs. On the other hand, the mouse model showed a difference of adhesion between the two cancer cell lines where MDA-MB-231 cells interacted in a larger amount compared to MCF-7. This result using the mouse *in vitro* BBB model is consistent with the results obtained with animal studies [29]. Hence, associated to *in vivo* experiments, the mouse model could represent a relevant BBB model to study brain metastases at a cellular and molecular level but is not suitable for human mechanistic studies. However, even if the phenotype of primary cells is closed to the *in vivo* situation, the primary cultures are expensive and time consuming [6,28]. Moreover, given the actual controversy over the use of animal models [30], particularly concerning the transposition of the results to the clinic, our experiments were secondly done on barrier models developed using human cells. Amongst human endothelial *in vitro* models, HUVECs are the most commonly cells used in oncology area for modeling the endothelial barriers and also to extrapolate to what happens at the level of the BBB [24,31,32]. However, characterization of HUVECS phenotype and its integrity is often lacking. In our study HUVECs did not show the required properties to be considered as a BBB model, notably they display a high permeability to the BBB integrity marker (LY) associated with a lack of continuous network of tight junctions at the cell-cell borders. Moreover adhesion experiment reveal that HUVECs are not able to reflect the cancer cell lines relative aggressiveness as MDA-MB-231 and MCF-7 cell lines had the same range of adhesion. In order to model the BBB *in vitro*, HUVECs were co-cultivated with cells from the NVU (GCs or pericytes). The co-culture system allowed a cross talk between the cell types through the secretion of soluble factors which induce and maintain the differentiation of ECs. Co-culture with GCs did not allow the establishment of the BBB properties in HUVECs but even led to an increase in Pe^LY^ associated with a lack of tight junction network. The result is consistent with the *in vivo* situation during the development of the BBB since pericytes are the first cell type recruited for the maturation of the BBB before astrocytes (the main cell type among GCs), which later have the role to maintain the BBB properties [33]. In line with this, co-cultivating HUVECs with pericytes decreased Pe^LY^ and induced the expression of tight junction proteins. The co-culture of HUVECS with pericytes, not previously described in the literature, had the aim to check if HUVECs were able to answer to differentiated factors secreted by pericytes. In this condition the results indicate that the induction of restrictive properties was not complete as the Pe^LY^ was still slightly high (0.96 ± 0.12 x 10^−3^ cm/min) and correlated with a discontinuous tight junctions network. The HUVECs models (solocultivated or co-cultivated with GCs) are commonly used in broad area of research (to study cancer, inflammation, Alzheimer’s disease…) to extrapolate what happened at the BBB level [23,24,31,34–36]. In the BBB field, the HUVEC model is recognized as not being reliable model to study the BBB. Our results emphasize this fact, actually as HUVECS have already a specific state of differentiation, co-cultivating them with NVU cells modify their phenotype but does not induce real BBB properties.

The BLECs model, recently published [11,12], obtained after co-culture of CD34^+^-ECs with pericytes presented a low Pe^LY^ (0.58 ± 0.07 x 10^−3^ cm/min) and continuous tight junction network at cellular borders. Furthermore, MDA-MB-231 and MCF-7 showed a significantly different adhesion on the BLECs model, correlating with the relative aggressiveness of the two cancer cell lines. Moreover, the use of additional breast cancer cell lines, demonstrate that the BLECs model is able to transcribe *in vitro* the heterogeneity of behavior the breast cancer cells. Hence, the capacity of adhesion of breast cancer cells is in line with the molecular subtype of cancer to which they belong. A cell line representative of an aggressive breast cancer adhere in a large amount compared to a cell line considered to not have a high brain metastatic behavior which adhere in a significant lower amount. As adhesion is required but not enough to reach the brain parenchyma, the BLECs were plated on 3-μm size pore filter to allow the study of the transmigration. The results obtained have revealed a differential of transmigration between the cancer cell lines in accordance with their described metastatic properties and in line with the adhesion results. Taking together, these results highlight the potential use of the human BLECs model as a tool to identify and characterize the pro-metastatic features of breast cancer cells and better understand their brain tropism.

At the molecular level, the glycosylation of breast cancer cells seems to be involved in the adhesion process. However as the adhesion is reduced but not completely inhibited, other protagonists seems to be implicated in the interaction processes.

The BLECs model will enable us to study the cellular and molecular mechanisms involved in early interaction steps between breast cancer cells and BBB ECs preceding brain metastasis formation. Additional experiments are necessary to identify more precisely the involvement of the glycosylation in interaction processes between the breast cancer cells and the BBB.

Our approach will help to better understand the brain tropism of breast cancer cells and will enable to analyze the breast-to-brain pro-metastatic features of breast cancer cells in order at least to identify novel therapeutic targets to block the brain metastases initiation at the BBB level.

## Supporting Information

S1 Fig Morphology of adherent MDA-MB-231 on endothelial cells. Visualization of adherent MDA-MB-231 on Endothelial cells after 2h of co-incubation. MDA-MB-231 were loaded with fluorescent CellTracker^™^ (green). Nuclei are stained with Hoechst (blue). Bar = 25 μm. HUVECs: Human Umbilical Vein Endothelial Cells; BLECs: Brain Like Endothelial Cells
(TIF)

S2 Fig BBB properties and cancer cells adhesion assay on HUVECs co-cultivated with NVU cells. (A) Visualization of tight junctions of HUVECs after co-culture with GCs. The HUVECs presents a discontinuous staining of tight junction proteins ZO-1 (left panel) and no Claudin-5 (right panel) associated with a high Pe^LY^ of 3.92 ± 1.04 x 10^−3^ cm/min. Interruption are indicated by white arrows. Nuclei are stained with Hoechst, bar = 50 μm. (B) Visualization of tight junctions of HUVECs after co-culture with pericytes. The HUVECs presents a discontinuous staining of tight junction proteins ZO-1 (left panel) and Claudin-5 (right panel) associated with a Pe^LY^ of 0.96 ± 0.12 x 10^−3^ cm/min. Interruption are indicated by white arrows. Nuclei are stained with Hoechst, bar = 50 μm.
(TIF)

## Abbreviations

BBB: Blood-Brain Barrier
BBCECs: Bovine Brain Capillary Endothelial Cells
bFGF: basic Fibroblast Growth Factor
BLECs: Brain Like Endothelial Cells
BSA: Bovine Serum Albumin
CD34^+^-ECs: Endothelial cells CD34^+^ stem cells-derived
CS: Calf Serum
DMEM: Dulbecco’s Modified Eagle’s Medium
ECs: Endothelial Cells
FCS: Fetal Calf Serum
GCs: Glial Cells
HS: Horse Serum
HUVECs: Human Umbilical Vein Endothelial cells
MBCECs: Murine Brain Capillary Endothelial Cells
NGS: Normal Goat Serum
NVU: NeuroVascular Unit
PBS: Phosphate Buffered Saline Calcium
Pe^LY^: Lucifer Yellow Endothelial Permeability
PPMP: DL-*threo*-1-Phenyl-2-palmitoylamino-3-morpholino-1-propanol
RH: Ringer Hepes
VEGF: Vascular Endothelial Growth Factor
ZO 1: Zonula Occludens 1
