# Supplementary figures and images for: Selection of a Relevant In Vitro Blood-Brain Barrier Model to Investigate Pro-Metastatic Features of Human Breast Cancer Cell Lines
Source: PLoS One. 2016 Mar 9;11(3):e0151155. doi: 10.1371/journal.pone.0151155 (PMC4784983; doi:10.1371/journal.pone.0151155)

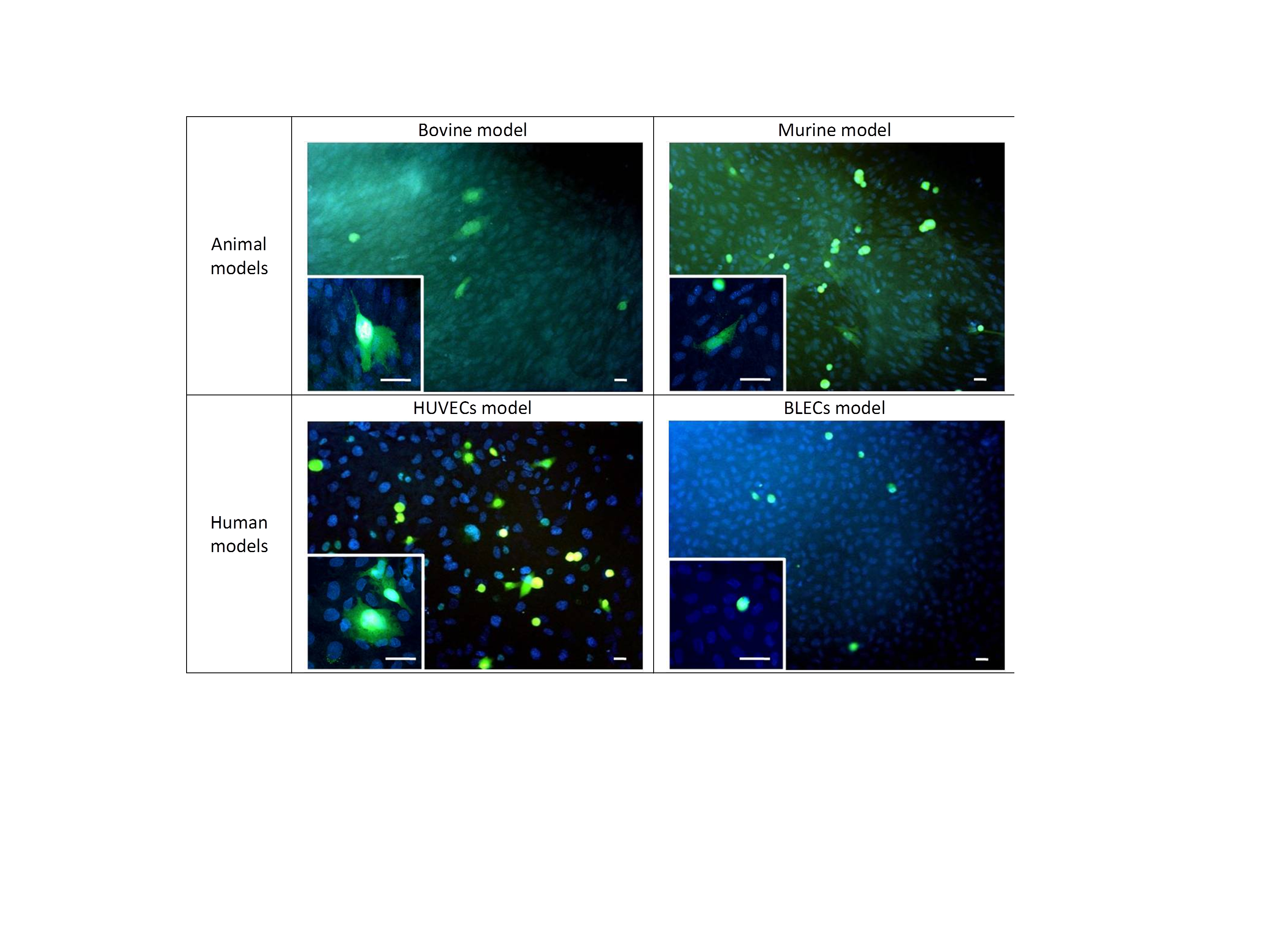

Supplement: S1 Fig — Visualization of adherent MDA-MB-231 on Endothelial cells after 2h of co-incubation. MDA-MB-231 were loaded with fluorescent CellTracker™ (green). Nuclei are stained with Hoechst (blue). Bar = 25 μm. HUVECs: Human Umbilical Vein Endothelial Cells; BLECs: Brain Like Endothelial Cells (TIF) [file pone.0151155.s001.TIF]

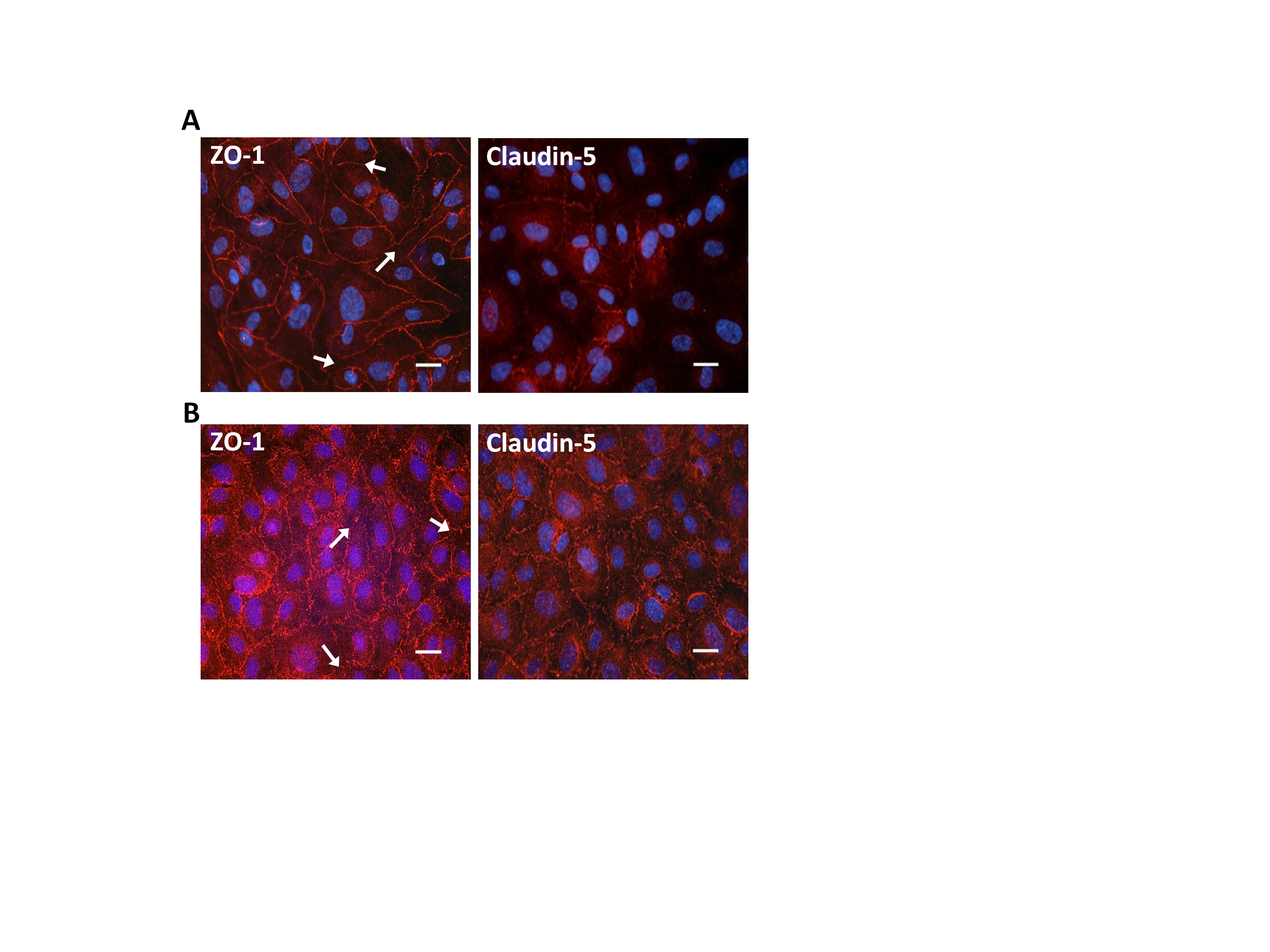

Supplement: S2 Fig — (A) Visualization of tight junctions of HUVECs after co-culture with GCs. The HUVECs presents a discontinuous staining of tight junction proteins ZO-1 (left panel) and no Claudin-5 (right panel) associated with a high PeLY of 3.92 ± 1.04 x 10−3 cm/min. Interruption are indicated by white arrows. Nuclei are stained with Hoechst, bar = 50 μm. (B) Visualization of tight junctions of HUVECs after co-culture with pericytes. The HUVECs presents a discontinuous staining of tight junction proteins ZO-1 (left panel) and Claudin-5 (right panel) associated with a PeLY of 0.96 ± 0.12 x 10−3 cm/min. Interruption are indicated by white arrows. Nuclei are stained with Hoechst, bar = 50 μm. (TIF) [file pone.0151155.s002.TIF]
